# Lipid bilayer composition influences small multidrug transporters

**DOI:** 10.1186/1471-2091-9-31

**Published:** 2008-11-25

**Authors:** Kalypso Charalambous, David Miller, Paul Curnow, Paula J Booth

**Affiliations:** 1 Department of Crystallography, Birkbeck College, University of London, London WC1E 7HX, UK; 2 Department of Biochemistry, University of Bristol, Bristol BS8 1TD, UK

## Abstract

**Background:**

Membrane proteins are influenced by their surrounding lipids. We investigate the effect of bilayer composition on the membrane transport activity of two members of the small multidrug resistance family; the *Escherichia coli *transporter, EmrE and the *Mycobacterium tuberculosis*, TBsmr. In particular we address the influence of phosphatidylethanolamine and anionic lipids on the activity of these multidrug transporters. Phosphatidylethanolamine lipids are native to the membranes of both transporters and also alter the lateral pressure profile of a lipid bilayer. Lipid bilayer lateral pressures affect membrane protein insertion, folding and activity and have been shown to influence reconstitution, topology and activity of membrane transport proteins.

**Results:**

Both EmrE and TBsmr are found to exhibit a similar dependence on lipid composition, with phosphatidylethanolamine increasing methyl viologen transport. Anionic lipids also increase transport for both EmrE and TBsmr, with the proteins showing a preference for their most prevalent native anionic lipid headgroup; phosphatidylglycerol for EmrE and phosphatidylinositol for TBsmr.

**Conclusion:**

These findings show that the physical state of the membrane modifies drug transport and that substrate translocation is dependent on *in vitro *lipid composition. Multidrug transport activity seems to respond to alterations in the lateral forces exerted upon the transport proteins by the bilayer.

## Background

Multidrug membrane transport proteins are very effective in antibiotic resistance as they pump drugs across bacterial membranes and out of cells. The family of small multidrug (SMR) transporters are the smallest known multidrug transport proteins [1], consisting of 4 transmembrane α helices [2-4]. The mechanism of multidrug transport is not understood in detail and SMR proteins provide an opportunity to probe the process in greater depth. Lipid composition and global properties of the lipid bilayer play key roles in membranes, often actively modifying the function of membrane proteins. Multidrug transporters themselves bind a variety of substrates and thus, flexibility in the transport protein binding pocket may be reflected in sensitivity to their surrounding lipids. In particular the proteins are likely to be sensitive to global lipid bilayer properties and the forces exerted on them by their surrounding lipids. Here, we investigate SMR proteins from two common pathogens; EmrE from *Escherichia coli *(*E. coli*)and TBsmr from *Mycobacterium tuberculosis *(*M. tuberculosis*). We focus on the influence of lipid bilayer composition on SMR protein function.

SMR proteins are proton, drug antiporters and extrude a variety of hydrophobic, cationic substrates through an exchange of the substrate and proton at a Glu residue (E14 for EmrE) [5]. Substrate binding and transport is thus pH dependent as it is affected by the protonation state of this Glu residue, the *pK*_*A *_of which has been estimated as 8.5 for E14 in EmrE [6]. EmrE confers resistance to a variety of molecules, including ethidium, methylviologen (MV), tetraphenylphosphonium (TPP) and tetracycline. TBsmr has 41% sequence identity to EmrE and transports ethidium and MV, but cannot transport TPP [7]. EmrE is the best characterised family member and seems to function as a dimer [4,8-13].

EmrE is found in the bacterial inner membrane of gram negative *E. coli*, the major constituents of which are phosphatidylethanolamine (PE) lipids, followed by anionic phosphatidylglycerol (PG) with a smaller proportion of cardiolipin. *M. tuberculosis *are classified as acid fast bacteria, as the high mycolic acid content of their cell walls is responsible for their resistance to acids that are used during staining procedures and results in poor staining compared to gram negative or positive bacteria. Despite differences between the cell walls of *E. Coli *and *M. tuberculosis*, TBsmr of the latter bacteria also resides in a membrane dominated by PE lipids, but the second major lipid constituent is anionic phosphatidylinositol (PI) lipid and then cardiolipin [14,15]. Here, we investigate the influence of lipid composition on MV transport by EmrE in defined lipid-bilayer vesicles with a PC background. Dioleoyl PC lipids with C18 chains each with one unsaturated bond (DOPC) form fluid lamellar bilayers, while the corresponding DOPE lipid alone forms non-lamellar phases as it induces monolayer curvature towards the aqueous phase. Increasing the proportion of DOPE increases the curvature elastic stress of the bilayer, which is also accompanied by an increase in the lipid chain lateral pressure [16-18]. These effects have been shown to affect the insertion, folding and function of membrane proteins [19-24]. In addition, we have previously shown that the DOPE content of DOPC/DOPE bilayers affects the activity of EmrE [25]. This reflects PE altering the overall properties and lateral pressure profile of the bilayer. We also found preliminary evidence for a role of the anionic lipid PG in enhancing activity. Here, we extend our work and investigate the effects of PE and PG on the activity of EmrE and TBsmr. In such a systematic study of lipid effects, we use synthetic lipids with defined chains as opposed to the native lipids, which for example in the case of *E. Coli *PE are a mixture of different chain lengths and saturation.

EmrE and TBsmr can be reconstituted into *E. coli *lipid vesicles and transport MV, but TBsmr transports less well than EmrE. We measure substrate transport in different lipid conditions. Optimisation of an established radioactive assay [8,26,27] enables us to compare the lipid influence on MV transport by the two transporters with different native lipid composition; EmrE from gram negative *E. coli *and TBsmr from acid-fast *M. tuberculosis*.

## Results

### MV transport

EmrE and TBsmr were purified into n-dodecyl-β-d-maltoside (DDM), as His-tagged protein following overexpression in *E. coli *according to previous methods [7]. Protein yields were ~1 mg for EmrE and 2.5 mg of TBsmr per litre of culture at > 95% purity.

Reconstitution and assay conditions were optimised using EmrE and *E. coli *lipid vesicles. Transport activity was quantified using pH driven transport of radiolabelled, ^14^C, MV a method that has been extensively applied to EmrE [8]. Previous reconstitution procedures [28] were optimised to maintain a pH gradient across the liposome bilayer during the transport measurement. EmrE was purified into DDM, but was then exchanged into octyl-β-D-glucopyranoside (OG) prior to reconstitution into lipid vesicles. Although EmrE is less stable in OG, this detergent is easier to remove from the bilayer than DDM (following reconstitution into lipid vesicles) as OG has a higher critical micelle concentration (CMC) than DDM (0.53% or 18 mM for OG as opposed to 0.009% or 0.17 mM for DDM). Thus this initial exchange of the protein to OG reduces proton leakage through the lipid bilayer enabling a proton gradient to be maintained which can drive MV translocation over minutes. The amount of OG remaining after reconstitution into *E. coli *lipid vesicles was 0.3 mM (well below the CMC of 18 mM and leaving a lipid:OG ratio 430:1). If EmrE was reconstituted directly from DDM into lipid vesicles (i.e. omitting the initial transfer to OG), 0.09% (1.8 mM) of DDM remained in *E. coli *protein-containing lipid vesicles. This is above the CMC of 0.009% and resulted in leaky lipid vesicles that could not maintain a pH gradient. The OG reconstitution procedure used here also involved only partial pre-saturation of the lipid vesicles by OG to help maintain bilayer integrity, but which also meant that <50% of the protein reconstituted. For example, 50 μg of EmrE was initially mixed with *E. coli *lipid vesicles but only 21 μg were found to be associated with the vesicles after reconstitution. Since SMR proteins are more unstable in OG compared to DDM, the proteins were immediately reconstituted into lipid vesicles following detergent exchange into OG.

**Figure 1:**
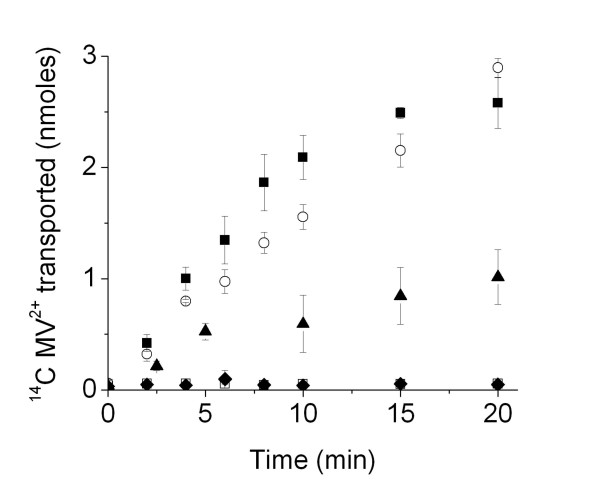
**MV transport by EmrE**. Radiolabelled, ^14^C MV^2+ ^transport into *E. coli *lipid vesicles by EmrE over time. EmrE reconstituted into *E. coli *lipid vesicles extruded to (■), 50 nm; (○), 200 nm and (▲), sonicated lipid vesicles. Control data is shown for (□), absence of a pH gradient and (◆), absence of protein in 50 nm lipid vesicles. Errors are shown as first standard deviation of 3 measurements on different protein preparations (with each value used for each protein preparation, for each data point shown, also being the average of 3 measurements on that particular preparation).

EmrE was reconstituted, via OG, into *E. coli *lipid vesicles that were initially prepared by sonication or extrustion to give differing sizes (50 and 200 nm in diameter). As shown in figure 1 increased assay reproducibility is observed when vesicles were formed by extrusion rather than sonication. In addition 50 nm diameter lipid vesicles were chosen over 200 nm lipid vesicles, since it was possible to determine both a linear regime of transport to determine an "initial rate of transport" as well as saturation of transport (by loss of pH gradient or substrate accumulation). The initial rate of MV transport by EmrE in 50 nm *E. coli *lipid vesicles was ~250 nmol. min^-1^. mg^-1^.

In summary, reconstitution of OG solubilised SMR proteins in OG pre-saturated lipid vesicles, gave tight lipid vesicles with low proton leakage and enabled linear MV transport to be observed in lipid vesicles over several minutes and thus initial rates to be determined. Radiolabelled MV transport provides a quantitative measure of EmrE transport activity. Activities over specified range of lipid conditions were quantified for EmrE and TBsmr by MV transport, as MV is a substrate for both proteins.

#### Lipid dependence of EmrE and TBsmr by MV transport

**Figure 2:**
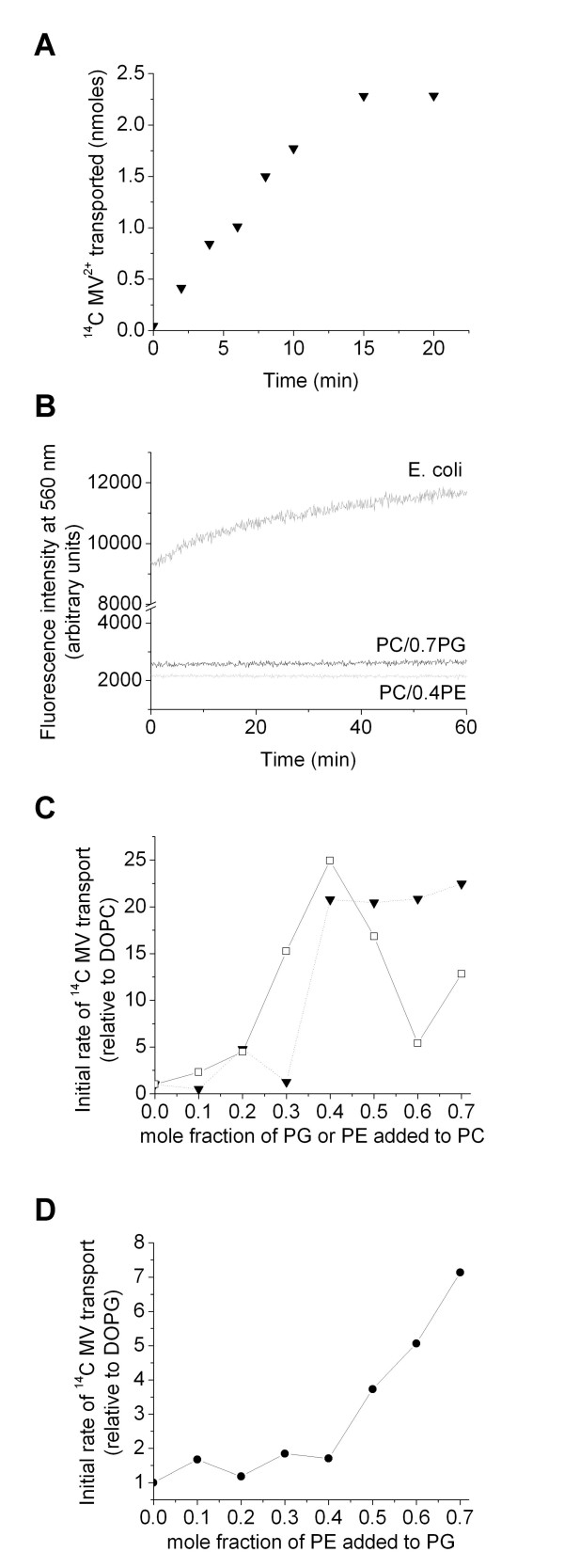
**(a) ^14^C MV^2+^transport by EmrE into vesicles of a defined mixture of lipids; DOPC/DOPG with 0.7 mole fraction DOPG**. Data are raw transport data, not corrected for the amount of protein associated with the lipid vesicles. (b) Fluorescence intensity over time of the pH sensitive dye, carboxyfluorescein, incorporated inside lipid vesicles, illustrating the maintenance of the pH gradient over time in protein-containing vesicles of varying lipid composition: *E. coli *lipids (upper dark grey trace); DOPC/DOPG with 0.7 mole fraction DOPG (middle black trace) and DOPC/DOPE lipid vesicles with 0.4 mole fraction DOPE (lower light grey trace). The fluorescence intensities in the lower two traces only increase by ~5% over 60 mins, which is within the noise of the data. A ~30% increase in fluorescence intensity occurs in the upper E. coli trace over 60 mins (~9% over 10 mins). (c) Initial rate of ^14^C MV^2+ ^transported by EmrE as a function of lipid composition in DOPC lipid vesicles. (□), DOPC/DOPE (▼), DOPC/DOPG. These initial rates are determined from the linear region of the MV transport data and additionally corrected for the amount of protein associated with the lipid vesicles. Thus, the point (▼) at 0.7 for DOPC/DOPG is determined from figure 2a. Data points are joined for clarification. (d) Initial rate of ^14^C MV^2+ ^transported by EmrE as a function of DOPE mole fraction in DOPG/DOPE lipid vesicles. All assays were carried out with 42 μM ^14^C MV^2+^. Data in figures 2c and d are initial rate data, corrected for the amount of protein, thus are per mg of EmrE. For comparison, they are shown relative to the rate in DOPC lipid vesicles in (b) or the rate in DOPG in (c) (i.e. with the relative rate for DOPC or DOPG being 1). Data in (a) are the average of for triplicate measurements for one protein sample (see methods), with the errors being smaller than the data points. Data in b and c are determined by linear fits to the initial rate data, as for example in (a); the errors from these fits are also smaller than the data points.

MV transport by EmrE was investigated in lipid vesicles of PC/PE, PC/PG and PG/PE. As an example, figure 2a shows raw data for pH-driven transport of MV into PC/PG lipid vesicles containing EmrE. We investigated maintenance of the proton gradient in the different lipid vesicles using a pH sensitive dye. Proton leakage has previously been shown to be unaffected by changes in membrane properties such as fluidity and lateral pressure induced by PE [29] and has been found to be particularly low in PE and anionic lipid containing membranes [30,31]. Figure 2b shows there was very little change in the fluorescence of the pH sensitive dye, carboxyfluorescein, enclosed within EmrE-containing lipid vesicles. The pH gradient was well maintained in all synthetic lipid mixtures during the time period (~10 mins) over which MV transport was linear with time. Good maintenance of the pH gradient (i.e. < 5% change in dye fluorescence) was observed over 60 mins for all PC/PE, PC/PG and PG/PE EmrE-containing lipid vesicles used here (EmrE being incorporated exactly as for the transport assays), apart from 100% DOPC lipid vesicles. A larger increase of about 25% over 60 mins (but still only ~6% over the 10 mins transport rate measurement period) was observed in DOPC lipid vesicles containing EmrE. This indicates a small, continual reduction in the pH gradient across these vesicles thus MV transport is probably slightly underestimated in this particular lipid composition. The initial fluorescence intensity of 10 μM carboxyfluorescein within the EmrE-lipid vesicles of all PC, PG and PE compositions varied by <15% (see figure 2b for two examples; PC/PG and PC/PE), indicating that the magnitude of the pH gradients was similar in all lipid compositions. A higher fluorescence intensity (see figure 2b) was however observed for *E. coli *lipid vesicles containing EmrE together with a gradual, non-linear increase in the fluorescence of ~30% over 10 mins, showing the pH gradient is poorly maintained in *E. coli *lipids. Despite this reduction of pH gradient, *E. coli *lipids showed higher rates of transport (~20-fold greater than in DOPC), thus still indicating an effect of lipid composition on transport activity.

In order to compare transport over a range of lipid compositions, the initial rates (obtained from the linear region of a graph such as figure 2a) were determined per mg of EmrE associated with the lipid vesicles. The protein concentrations used in the assays were quantified using Western blots following colorimetric detection. The amount of protein associated with the lipid vesicles was found to be independent of lipid composition. In order to eliminate variations in initial rates due to different protein preparations, the same thawed protein sample was used for comparisons of rates across each lipid series; for example, a protein sample was exchanged from DDM into OG, and aliquots of this one sample used for all PC/PG measurements. We also only compare relative increases in initial rates for the same protein sample.

Figure 2c shows the initial rate of MV transport increases, when increasing amounts of PE or PG are incorporated in DOPC lipid vesicles. The initial rates per mg EmrE are plotted relative to the rate in DOPC, with the actual initial rate values being given in table 1. Figure 2c shows there is an abrupt increase with PG at around 0.4 mole fraction PG and with a mole fraction of 0.7 the rate is ~22-fold greater in PC/PG lipid vesicles than in PC alone. The initial MV rate also increases with PE, in PC/PE with an apparent maximum rate at about 0.4 mole fraction PE in PC/PE, which is about 25-fold greater than the rate in DOPC. At higher PE mole fractions (~0.6–0.7) there may be some phase separation of PE. Figure 2d shows that there is also an increase in MV rate as the PE content of PG/PE lipid vesicles is increased (rates shown relative to that in DOPG). PG/PE represents the most favourable environment in terms of initial MV transport rate: there is a 7-fold increase in rate over DOPG alone, when 0.7 mole fraction of PE is present, which in addition to the fact that the rate in DOPG is ~6 times that in DOPC, means that PG/0.7 PE gives over 40-fold enhancement in MV rate over DOPC alone (see table 1). The effect of PG on MV transport is not a straightforward, additive effect of the PC and PG mixtures but seems to be more complex, since larger rates are noted for PC/PG mixtures than in either PC or PG alone. Thus, the initial rate of MV transport increases to a maximum in PC/PG with 0.7 mole fraction PG, where it is ~22-fold greater than in DOPC. The rate enhancement in DOPG is however, less than this, being only ~6-fold greater than in DOPC.

**Table 1:** Initial rate of ^14^C MV^2+ ^transported by EmrE and TBsmr as a function of lipid composition

	**EmrE** **Initial rate of ^14^C MV^2+ ^transport (nmol. min^-1^. mg^-1^)**			**TBsmr** **Initial rate of ^14^C MV^2+ ^transport (nmol. min^-1^. mg^-1^)**				
	
**Mole fraction of added lipid^a^**	***DOPC/DOPE*** ***(PE added to PC)***	***DOPC/DOPG*** ***(PG added to PC)***	***DOPG/DOPE*** ***(PE added to PG)***	***DOPC/DOPE*** ***(PE added to PC)***	***DOPC/DOPG*** ***(PG added to PC)***	***DOPG/DOPE*** ***(PE added to PG)***	***DOPC/SoyPI*** ***(PI added to PG)***	***SoyPI/DOPE*** ***(PE added to PI)***
0	14	14	81	13	13	58	13	53
0.1	32	7	135	7	7	23	45	ND
0.2	62	67	95	7	22	49	50	86
0.3	213	18	149	12	18	26	43	ND
0.4	349	291	138	55	30	26	261	316
0.5	235	286	301	44	30	29	251	ND
0.6	76	292	409	22	59	47	104	331
0.7	179	315	577	33	28	39	94	ND

a The mole fraction is that of the second lipid; for DOPC/DOPE it refers to the mole fraction the DOPE that is added

**Figure 3:**
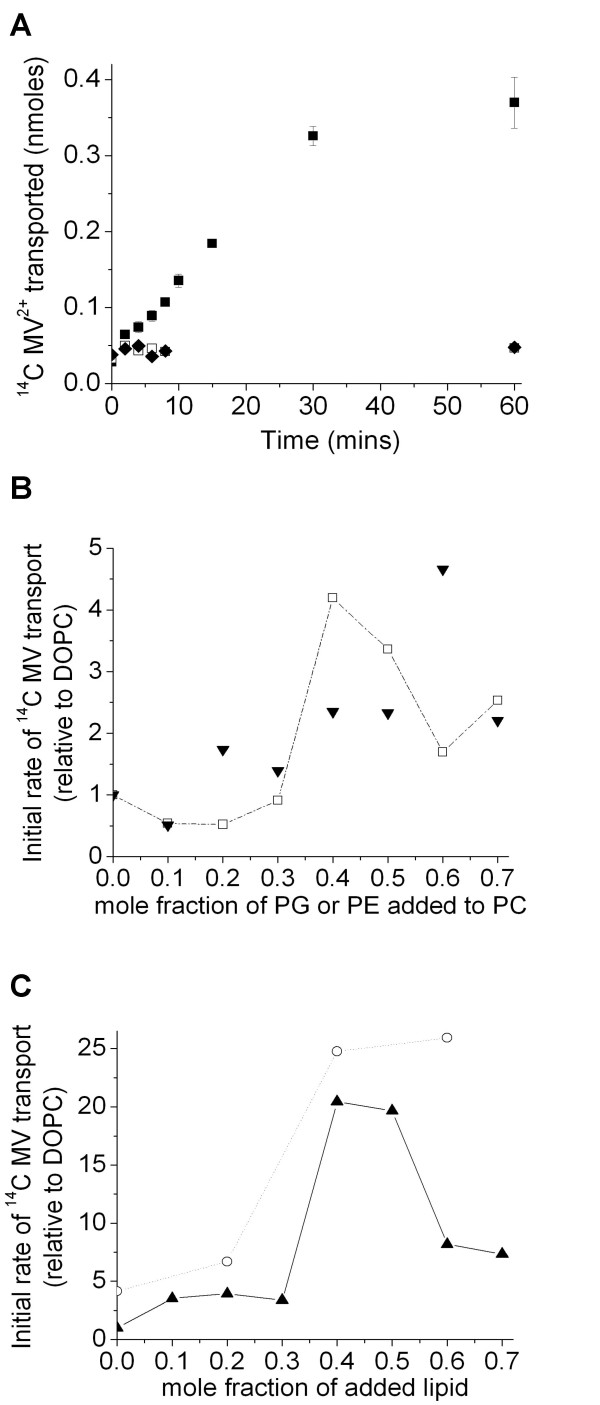
**MV transport by TBsmr**. (a) ^14^C MV2+ transport into (■) 50 nm extruded *E. coli *lipid vesicles over time; control data with (□) no TBsmr present; (◆), no pH gradient. Initial rate of ^14^C MV transport as a function of lipid composition into (b) (□), DOPC/DOPE; (▼), DOPC/DOPG and (c) (▲) DOPC/soyPI; (○), soyPI/DOPE (the second lipid in each case is the mole fraction on the x axis). Data points are joined to clarify trends. Data in figures 3b and 3c are initial rate data, corrected for the amount of protein (thus are per mg of TBsmr) and shown relative to the rate in DOPC lipid vesicles. There is a larger pH gradient than for EmrE (pH 9 on the outside of the liposome for TBsmr, but pH 8 for EmrE and pH 7 inside for both proteins). As for EmrE, 42 μM ^14^C MV^2+ ^was used in the TBsmr measurements.

TBsmr was less active than EmrE in the lipids used here thus the amount of protein and the pH gradient were increased by raising the pH from 8 to 9 outside the lipid vesicles (and keeping the interior pH at 7.5). Figure 3a shows that linear transport was observed over longer times than for the EmrE experiments, but that less MV was incorporated into the lipid vesicles even with the larger pH gradient (*cf *figure 1). The rate of MV transport by TBsmr was ~50 nmol. min^-1^. mg^-1 ^in *E. coli *lipids (with pH 9 outside the lipid vesicles), which is approximately 5 fold slower than that of EmrE of ~250 nmol. min^-1^. mg^-1 ^(with pH 8 outside). MV transport by TBsmr was investigated in the same PC, PE and PG lipid systems as for EmrE. Additionally since the native TBsmr membrane contains PI (as opposed to PG) transport in DOPC/Soy PI and Soy PI/DOPE lipid vesicles was also investigated. Figure 3b and table 1 show that the rate of MV transport by TBsmr increased with increasing PE or PG in PC lipid vesicles, with a maximum at about 0.4 mole fraction PE, similarly to EmrE (see figure 2c) but the overall increase is only about 4-fold, and lower than that for EmrE. This smaller dependence on lipid composition also masked any effect of PE in PG/PE lipid vesicles (see table 1). A larger increase in TBsmr activity was seen with increasing PI in PC/PI lipid vesicles, for which there was a maximum transport rate at about 0.4–0.5 mole fraction PI that was about 20-fold greater than the rate in DOPC (3c). The addition of PE to the anionic PI caused ~5-fold increase in PI/0.6 PE over DOPG or ~25-fold increase over DOPC (see figure 3c).

In summary, the activity of both EmrE and TBsmr increase with PE or anionic lipid, with the native PI lipid increasing the rate more than PG for TBsmr. The addition of DOPE to DOPC bilayers has the same effect on EmrE and TBsmr initial rates of transport. An increase in activity, which maximizes at 40% DOPE is observed followed by a gradual decrease above this DOPE percentage. In addition the largest increase in TBsmr activity is observed upon the incorporation of PI to DOPC lipids, a phenomenon mirrored upon the addition of DOPG to DOPC with respect to EmrE activity. Finally the largest initial rates are seen for PG/PE or PI/PE mixtures, respectively for EmrE or TBsmr.

#### Lipid dependence of EmrE substrate K_m_

**Figure 4:**
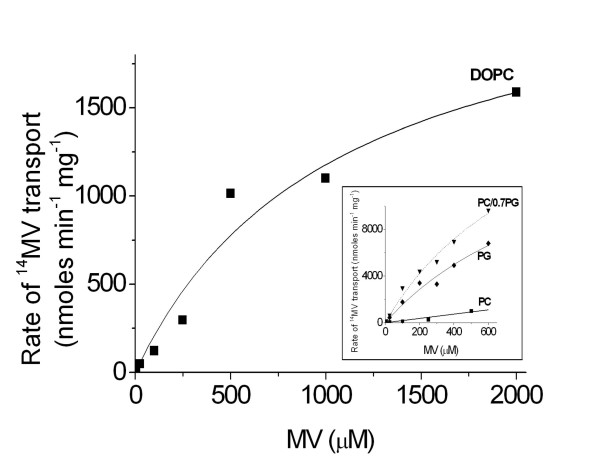
**Initial transport rate as a function of ^14^C MV^2+ ^concentration in DOPC**. Inset: data over 600 μM ^14^C MV^2+ ^for transport in (■), DOPC; (◆) DOPG and (▼) DOPC/DOPG with 0.7 mole fraction DOPG. Curves represent fits of the data to the Michaelis Menten equation.

K_m _and V_max _were estimated for MV transport by EmrE in the different lipid mixtures. Figure 4 shows the dependence of the EmrE initial transport rate on MV concentration in DOPC, which in a simple, Michaelis Menten model would saturate at some point; when all proteins are transporting. However, the curves did not saturate fully even at very high MV concentrations, >2 mM (and similarly for PC/PG mixtures; non specific MV binding being subtracted at each MV concentration in all cases). Such a high 2 mM concentration of MV corresponds to about 1 MV molecule per lipid and since the positive charges and overall polarity of MV means it can bind to phospholipid headgroups [32], MV will bind and significantly interfere with the liposome structure and lipid bilayer properties at this 1:1 ratio. Thus, the bilayer properties will be gradually altered as the MV concentration increases and at high MV concentrations will be significantly different to those of MV-free lipid vesicles. Since, bilayer composition and properties influences transport activity by EmrE, this means the bilayer itself is changing and is no longer inert during these MV measurements. The background, non-specific binding (or partitioning) of MV to the lipid vesicles increased with MV concentration, being ~30 times greater at 600 μM MV than at 42 μM MV. However, the background binding did not change over time at any MV concentration and thus does not represent passive diffusion of MV across the bilayer (data not shown).

The non-specific binding/partitioning of MV was found to depend on lipid composition. Twice as much MV partitioning was observed in 100% DOPG than in 100% DOPC (data not shown). The higher partitioning of MV into DOPG vesicles is most likely due to the negative charge of DOPG lipids attracting the positively charged MV. This effect could also partly explain the greater transport of MV into DOPG lipid vesicles by EmrE, since there will be a higher local concentration of MV present at the membrane surface or partitioned into the membrane. However, there must also be an additional effect, other than charge, since the highest rates of transport by EmrE are observed with mixtures of DOPC and DOPG, rather than pure DOPG. An overall decrease in MV associated with lipid vesicles was observed in PG/PE lipid vesicles as the proportion of PE increased up to 40% but no further variation was observed above 40% PE.

In spite of the lack of complete saturation (see figure 4), we estimated *K*_*m *_or V_max _for PC/PG mixtures after taking more data points up to 600 μM MV (see inset figure 4). No significant changes could be detected in K_m _(of ~1 mM) as the PG mole fraction was altered, but the V_max _increased with increasing PG, from 2 μmole.min^-1 ^in DOPC, to around 20 μmole.min^-1 ^in DOPC/DOPG mixtures or DOPG alone (see table 2). Thus, the presence of PG above a mole fraction of 0.5 causes an approximate 10-fold increase in V_max_, but no detectable change in *K*_*m*_. This shows that the 22-fold increase in initial rate upon addition of >0.4 mole fraction PG (see figure 2c and table 1) is reflected in an increase in V_max_.

**Table 2:** V_max _and K_m _for EmrE in DOPC/DOPG liposomes

**Mole fraction of DOPG added to DOPC**	**V_max _(μmole.min^-1^)**	**K_m _(mM)**
0	2 ± 2	1.1 ± 0.5
0.5	20 ± 2	0.9 ± 0.5
0.7	24 ± 6	1.0 ± 0.4
1	19 ± 6	1.2 ± 0.8

Errors are the standard deviation of the fit of the data to the Michaelis Menten curve)

MV transport data at high MV with PE present in the lipid vesicles (i.e. DOPC/DOPE or DOPG/DOPE) was too error prone for analysis and the data did not show the same trends with PE at high and low MV. The initial rate was approximately linear with MV, did not saturate and did not fit to Michaelis Menten kinetics. This suggests significant interaction of MV when PE is present and it was not possible to estimate *K*_*m *_or V_max _values.

## Discussion

We have found that lipids have significant influence on multidrug transport. The addition of DOPE or DOPG to a DOPC background is able to alter substrate transport by SMR proteins by over an order of magnitude. A comparison of two transporters, EmrE and TBsmr, from bacteria with dissimilar native lipid compositions, reveals commonalities in their lipid dependences.

### Lipid dependences of the SMR family of proteins

The native membranes of EmrE and TBsmr are of similar composition with PE lipids being the majority constituent closely followed by an anionic lipid. The most abundant anionic lipids in EmrE native membranes are PG lipids opposed to PI lipids found in native TBsmr membranes. The initial rate of MV transport at low MV concentrations proved a reliable method of comparing the lipid dependences of the two protein activities. MV uptake has been previously used to determine EmrE function and initial rate values ranging between 160 to 30,000 nmol.min^-1^.mg^-1 ^have been reported for EmrE reconstituted into *E. coli *vesicles[7,8,26,27]. The diverse values are attributed to variations in reconstitution methodologies, solubilisation conditions and lipid concentrations. Our initial rate values (of 250 nmol.min^-1^.mg^-1 ^for EmrE in *E. coli*) are in agreement with the lower end of this range of values. *In vivo *experiments have been utilised to identify SMR ligands [7,8] but limited kinetic characterisation has been undertaken. Here, we have investigated how varying lipid compositions, which mimic aspects of the *in vivo *environments, affect SMR transport *in vitro*.

The incorporation of PE into PC bilayers increases the initial rate of MV transport for both EmrE and TBsmr up to 0.4 PE mole fraction (see figures 2c and 3b). TBsmr is less active in DOPC lipid vesicles than EmrE and shows a smaller, 4-fold increase in transport rate with PE, compared to 20-fold for EmrE. Both proteins also showed rate increases when DOPG was added to DOPC; about 25-fold for EmrE and 5-fold for TBsmr. TBsmr showed a larger, 20-fold, increase in rate when its native PI lipid headgroup was added instead of DOPG (figure 3c). These results show that a fluid DOPC lipid bilayer structure imparts a degree of transport activity to SMR proteins. However, transport is suboptimal in DOPC and regardless of the lipid added to DOPC, an increase in transport rate is observed when the DOPC mole fractions falls below 0.6 (see figures 2c,d and 3b,c). This implies a threshold level of PC of ~0.6 mole fraction in binary lipid mixtures, above which transport is hindered. An increase in rate occurs upon addition of PE or an anionic lipid to PC. A faster transport rate still is seen in the absence of PC, but with a combination of PE and the anionic lipid. These are the dominant lipid types in the native membranes: PG/PE for EmrE and PI/PE for TBsmr, which give ~70-fold and 25-fold increases in initial rate over that in DOPC (which is not present in either native membrane). In this study, synthetic lipids with defined chain compositions were used rather than the mixtures of differing chain length and saturation present in native lipid compositions, thus these varying natural chains could also further enhance transport activity. The data for EmrE in PC/PG (figure 4) show that the increase is due to PG increasing the maximum transport rate, V_max_. The negative charge of PG or PI is likely to play a role in attracting the positively charged MV substrate. The larger increase in transport observed with PI over PG for TBsmr, presumably reflects a preferential interaction with its native inositol group.

### Lipid effects on membrane proteins

Membrane proteins are heavily influenced by their surrounding lipid environment. Specific lipid interactions have been identified with lipids either being seen tightly bound in X-ray structures or a certain lipid being essential for function [33]. Alternatively, generic bilayer properties can significantly influence protein function [23]. Key properties include a mis-match between the hydrophobic length of the protein and lipid bilayer as well as the elastic properties of the bilayer, which include curvature energy and lateral pressure. Thus, alamethicin channel formation and function has been directly linked to bilayer curvature in PC/PE bilayers [21,22], folding is also dependent on this curvature and lateral pressure in PC/PE systems [20]. Here we have shown that PE favours EmrE or TBsmr transport activity, in an anionic lipid background (PG for EmrE or PI for TBsmr). Although, the greatest influence on transport is the anionic lipid.

Lipids have also previously been shown to affect transport function but it is only recently that systematic studies have been undertaken to investigate their effect on integral membrane proteins. *E. coli *lactose permease is known to be affected by lipids [34], with initial rates of lactose showing similar dependences on PE as reported here for SMR proteins. The lactose rate increased upon addition of 0.5 mole fraction PE to PC. The effect of PG differed to that reported here, as 0.5 mole fraction PG did not increase the lactose rate, unlike the large 20-fold increase seen here for EmrE. PE has also been reported to affect the biogenesis, folding and topology of lactose permease and other transporters [35-37]. Other multidrug transporters of the major facilitator superfamily (MFS) including GabP, PheP and LmrP [38-40] have shown lipid dependencies for function. In the case of LmrP the replacement of PE for PC in the bilayer lead to significant alterations in both structure and function. Further studies on LmrP using PE-methylated moieties suggest that direct interactions between the PE headgroup and certain LmrP amino acids are directly involved in pH sensing required for substrate transport [41]. There are also some indications that multidrug transporters from other protein families are also dependent on lipid composition. In the case of the ATP binding cassette multidrug transporter, p-glycoprotein, drug binding has also been shown to be influenced by lipid environment [42].

## Conclusion

The data presented here highlight the importance of investigating the influence of the lipid environment on SMR protein activity. The addition of PE to PC bilayers has the same effect on both EmrE and TBsmr activity demonstrating that the lipid environment of homologous proteins affects their activity in the same manner. Overall, these data show that the use of sub-optimal lipid compositions can have a large effect on the transport kinetics and relative substrate affinities obtained during *in vitro *study of multidrug transport.

## Methods

Lipids were from Avanti Polar Lipids Inc.: DOPC, DOPG, DOPE, *E. coli *or soyPI, where the first three are pure synthetic lipids (with two C18 chains each with one unsaturated bond), and the last two are natural lipid extracts, containing a mixture of lipid chains lengths and saturation. *E. coli *lipids also contain a range of headgroups. Detergents DDM and OG were from Anatrace; anti c Myc alkaline phosphatase from Sigma-Aldrich; Ni-NTA agarose from QIAGEN, carboxyfluorescein from Molecular Probes and all other compounds were obtained from Sigma-Aldrich.

### Protein preparation

EmrE-His and TBsmr-His were cloned into pT7-7, transformed into *E. coli *TA15/pGP1-3 and overexpression (induced by heat shock) and purification were performed as previously described [3,5]. A 6 L volume of cells was disrupted by passage through a French ™pressure cell at 1000 psi (6900 kPa). Isolated membranes were solubilised at 4°C for at least 2 hours in 50 ml of solubilising buffer (20 mM Tris, pH 7.5, 100 mM NaCl, 10 mM 2-mercaptoethanol, and 1% (w/v) DDM). Insoluble material was removed by centrifugation at 35 000 g and 4°C. NaCl and imidazole were added to final concentrations of 350 mM and 15 mM respectively, and DDM solubilised protein was consequently incubated with 1.5 ml of Ni-NTA agarose (QIAGEN) for 1.5 h at 4°C. Non-specifically bound protein was removed by washing with 30 column volumes of 20 mM Tris pH 7.5, 400 mM NaCl, 15 mM imidazole, 0.1% DDM and 5 mM 2-mercaptoethanol, followed by 15 column volumes of the above buffer with the exception of 20 mM NaCl. Proteins were eluted with 5 column volumes of elution buffer (20 mM Tris, pH 7.5, 25 mM NaCl, 200 mM imidazole, 0.1% DDM and 5 mM 2-mercaptoethanol). Eluted protein was concentrated in an Amicon Ultra 50 000 MWCO centrifugal concentrator (Millipore) to approximately 1–2 mM as determined by A_280_, snap frozen in liquid N_2 _and stored at -80°C until required. Protein purity was determined by SDS-PAGE and Western blot analysis. His-tagged protein was used throughout. EmrE purified into DDM was assayed for activity by TPP binding, by radiolabelled TPP binding curves as well as isothermal titration calorimetry. The latter gave a dissociation constant, *K*_*d *_of 36 nM in 0.08% DDM with a binding stoichiometry of 0.48 TPP per EmrE, i.e. binding to dimer EmrE, with > 90% of the protein being active.

### Lipid vesicles

Lipid vesicles were prepared with 50 nm, 100 nm or 200 nm diameter by extrusion as previously [43,44]. Vesicles were also prepared by sonication.

### Transport assay

Transport assays were performed as previously described [8] using radiolabelled ^14^C MV^2+ ^(Sigma-Aldrich) (referred to as MV throughout). Protein was first exchanged from DDM into OG [28]: DDM-protein was incubated with 1.5 ml of Ni-NTA beads (pre-washed 1% OG and 20 mM Tris pH 7.5) for 1.5 h at 4°C; loaded onto a Pharmacia XK-16/20 glass column; washed 4-times with buffer containing 150 mM NaCl, 1% OG, 15 mM mercaptoethanol and 15 mM Tris pH 7.5 and then mixed with elution buffer (the previous buffer with 200 mM imidazole and 15 mM mercaptoethanol) and incubated at room temperature for 15 min. Eluted protein was concentrated by centrifugation at 4000 g using Amicon Ultra 50 000 MWCO centrifugal concentrator (Millipore). Protein in OG was immediately reconstituted into lipid vesicles (at a starting protein:lipid mole ratio of ~2900:1). Lipids were rehydrated (to 50 mg.ml^-1 ^in 150 mM NaCl, 15 mM Tris, pH 7.5) and sonicated or extruded to 50 nm, 100 nm or 200 nm as stated in the text. The vesicles were mixed with OG to a final concentration of 0.65% w/v, or a 25:1 molar ratio of lipid to OG. 75 μg of OG solubilised EmrE-His was added to 375 μl of these OG-lipid vesicles and OG was removed by a ~70 fold dilution into 25 ml of 190 mM NH_4_Cl, 15 mM Tris pH 7.5 followed by mixing at room temperature for 20 min. The protein-containing vesicles were centrifuged at 250 000 g for 60 min, at 25°C and the pellet resuspended in 100 μl of reconstitution buffer (190 mM NH_4_Cl, 15 mM Tris pH 7.5) and stored at -80°C. Protein-containing vesicles were thawed, resealed by a 20 s sonication step and transport was initiated by dilution of 3 μl of protein-containing vesicles into 200 μl of assay buffer (140 mM KCl, 5 mM MgCl_2 _and 10 mM Tricine) containing 42 μM ^14^C MV^2+^, at pH 8 for EmrE and pH 9 for TBsmr. At given times ranging between 0 and 20 min the reaction was stopped by dilution with 2 ml of ice cold assay buffer and protein-containing lipid vesicles were collected by filtration through Scheicher and Schull (0.2 μm) (Millipore, Watford, UK) filters. (Although the procedure commenced with extruded unilamellar vesicles, their size will have altered during reconstitution and the transport assay. We found, as previously reported [8], that 0.2 μm filters worked well and better than smaller pore sizes). Filters were placed into scintillation vials with 10 ml of Emulsifier-safe scintillation fluid (PerkinElmer) and radioactivity was measured in a TRI-CARB 2100TR Liquid Scintillation Counter. Experiments were carried out in triplicates.

The amount of OG and DDM remaining after reconstitution was determined by a phenol sulphuric acid carbohydrate assay [45]. For this assay, 50 μl of the protein-containing vesicles were added to 250 μl of 5% (w/v) phenol and 600 μl of concentrated sulphuric acid. This mixtures was incubated for 5 min and the absorbance was measured at 460 nm.

Maintenance of pH was monitored by incorporation of the pH sensitive dye carboxyfluorescein (Molecular Probes) inside protein-containing lipid vesicles. Protein-containing vesicles were formed as described above in the presence of 10 μM carboxyfluoroscein. 3 μl of protein-containing vesicles were diluted into 200 μl of assay buffer and thoroughly mixed by pipetting. Changes in carboxyfluoroscein fluorescence were monitored over 15 min, at 25°C by exciting the sample at 475 nm and measuring emission at 520 nm.

Initial transport rates of MV were determined from the gradient of the linear part of the MV accumulation versus time (e.g. figure 1a): over ~the first 6 mins for EmrE or 16 mins for TBsmr. Rates (apart from those for K_m _and V_max _determinations) were determined for 42 μM MV. All fitting was carried out using MicroCal Origin 6.0 software. Initial rates were calculated per mg of protein associated with lipid vesicles after reconstitution. Note that identical protein samples were used for each lipid series; protein aliquots were taken from the same protein sample exchanged from DDM into OG for all DOPC/DOPE measurements, or all from a separate protein sample for all DOPC/DOPG or DOPG/DOPE measurements.

The amount of protein was quantified on samples after the transport assay, on the same sample, from colorimetric development of Western blots [46], by densitometry using Quantity One^® ^software (BioRad). The actual amount of reconstituted protein was calculated by comparing band densities between protein-containing lipid vesicle samples, normalised to an aliquot of the same protein preparation which had been previously quantified (each Western always contained a band from a protein sample that had already been quantified on another gel, to enable normalising between gels). Protein-containing vesicle samples were run on 10% SDS denaturing gels and transferred to polyvinyl difluoride membranes prior to incubation with anti c Myc alkaline phosphatase (1:5000) (Sigma Aldrich) (the protein constructs contained a Myc epitope). Alkaline phosphate substrate solution, BCIP-NBT (Sigma Aldrich), was applied to the membrane as per manufacturer's protocol for protein visualisation and quantification. The BCIP-NBT detection system is based on the hydrolysis of 5-bromo-4-chloro-3 indolyl phosphate (BCIP) and the reduction of p-nitrobluetetrazolium chloride (NBT) producing a purple product, which can be colorimetrcally quantified. This Western blot method due to its high sensititivity (0.5 ng of substrate) [47,48]proved effective for quantifying the relatively small amounts of EmrE reconstituted by the method here, which was optimised to maintain a pH gradient rather than to optimise EmrE concentrations. The amount of reconstituted protein was also assessed by a modified Lowry method as well as following solvent extraction of protein and lipid. For the latter EmrE samples in lipid were solubilised using a 43:43:14 chloroform:methanol:sample (v/v) ratio and absorbance spectrum measured between 250 and 310 nm. EmrE standards of known concentration in DDM were used for calibration and lipid samples in the absence of EmrE were used to subtract any non-protein absorbance in the region from 250–310 nm.

In order to determine K_m _and V_max _MV transport assays were performed with different concentrations of ^14^C MV at three different lipid mole fractions for each lipid series: DOPC/DOPE, DOPC/DOPG and DOPC/DOPG. MV concentrations were between 5 μM and 2 mM MV (spiked with 5% ^14^C MV^2+^). The extent of non-specific binding of MV was determined in control experiments in protein-free lipid vesicles (for all lipid compositions). This non-specific binding was corrected for in the binding curves shown. At 42 μM MV this background binding was significantly lower than the specific binding and transport. 42 μM MV corresponds to: ~250:1 mole ratio MV to protein and ~50:1 lipid: MV; 600 μM MV to: ~4000:1 MV to protein, and 3:1 lipid: MV.

## Abbreviations

CMC: Critical Micellar Concentration; DDM: n-dodecyl-β-d-maltoside; DOPC: 1,2-dioleoyl-sn-glycero-3-phosphocholine; DOPE: 1,2-dioleoyl-sn-glycero-3-phosphoethanolamine; DOPG: L-α-1,2-dioleoyl-sn-glycero-3-phosphoglycerol; *E. coli*: *Escherichia coli*; EmrE: *Escherichia coli *small multidrug transporter; *M. tuberculosis*: *Mycobacterium tuberculosis*; MV: methylviologen; OG: Octyl-β-D-glucopyranoside; PC: phosphatidylcholine; PE: phosphatidylethanolamine; PG: phosphatidylglycerol; PI: phosphatidylinositol; SDS: Sodium Dodecylsulfate; SMR: small multidrug resistance; TBsmr: *Mycobacterium tuberculosis *small multidrug transporter.

## Authors' contributions

KC devised and performed all experimental work. DM assisted with protein preparations, protein activity assays, data analyses and experiment design. PC advised on experimental design, assisted with data analyses and performed background experimental work. KC, DM and PC helped to draft the manuscript. PJB conceived the study, designed experimental approaches and wrote the manuscript.
